
JOURNAL INFORMATION
==============================
NLM Title Abbreviation: Gastroenterol Rep (Oxf)
Journal ID: Gastroenterol Rep (Oxf)
NLM Journal ID: 101620508
Journal ID: gastro

Gastroenterology Report

EISSN: 2052-0034
Publisher: Oxford University Press

ARTICLE INFORMATION
==============================
PMCID: PMC4976686
DOI: 10.1093/gastro/gow027
Article ID: gow027
Article version: 1
Subjects: Chinese Abstracts

Chinese Abstracts – Volume 4 Issue 3*

*This article abstract was originally published in English. This translation has not been verified and should not be relied upon—it is provided for reference purposes only. The Publishers have not checked this translation and accept no liability for completeness or accuracy of this translation or the use of this translation for whatever purpose. This translation may be incomplete and inaccurate in whole or in part. If you need to rely upon a translation of this abstract, a professional human translator should be engaged to supply an accurate translation of the original English. When referencing articles from this journal, please always refer to the original English version, rather than a translated equivalent.

Print publication date: 2016 Aug
Electronic publication date: 2016 Aug 5
Volume: 4
Issue: 3
First page: C1
Last page: C3
Copyright: © The Author(s) 2016. Published by Oxford University Press and the Digestive Science Publishing Co. Limited.
Copyright year: 2016
License: This is an Open Access article distributed under the terms of the Creative Commons Attribution License (http://creativecommons.org/licenses/by/4.0/), which permits unrestricted reuse, distribution, and reproduction in any medium, provided the original work is properly cited.
License URL: https://creativecommons.org/licenses/by/4.0/

Page count: 3

==============================
OXFORD

*Gastroenterology Report*, 2016, 1–3

doi: 10.1093/gastro/gow027

### Chinese Abstracts – Volume 4 Issue 3*

### Review （综述）

### Pelvic autonomic nerve preservation in radical rectal cancer surgery: changes in the past three decades
### 直肠癌根治术中的盆腔自主神经保护：30年变迁

[摘要] 全直肠系膜切除（TME）理念及微创技术（如腹腔镜结直肠手术和机器人手术）的出现大大提高直肠癌的外科疗效。然而，术后排尿功能和性功能障碍率仍然较高，从而给患者造成巨大的痛苦，同时也会给手术医生带来困扰。新辅助和辅助放疗应用的增多也带来了更多的功能问题。在本文中，我们对盆腔神经解剖的目前认识进行了评价，将盆腔神经分为肠系膜下动脉根部、侧盆壁及泌尿生殖器官周围等区域，探讨了在这些区域如何进行手术操作。同时，我们也讨论了过去 30 年不同手术方式（包括开腹、腹腔镜和机器人）的功能状况。

关键词：全直肠系膜切除术；盆腔自主神经保护；排尿功能障碍；性功能障碍

### Oesophageal and gastric varices: historical aspects, classification and grading: everything in one place
### 食管胃底静脉曲张的历史沿革、分类和分级

[摘要] 静脉曲张及其处理在门脉高压的治疗中极为重要。对于门脉高压中静脉曲张的处理，目前的各种指南都趋于统一。然而，静脉曲张的分类和分级在世界各地却各不相同。本文重点讨论了静脉曲张分类和分级系统的历史沿革和应用现状，并辅以自制的图表，力图令读者对门脉高压和静脉曲张有一个更加深入且清晰的认识。

关键词：食管静脉曲张；胃底静脉曲张；分类；门脉高压

### Heterotopic gastric mucosa in the anus and rectum: first case report of endoscopic submucosal dissection and systematic review
### 肛管直肠的异位胃黏膜：首例内镜黏膜下剥离术治疗报道和系统综述

[摘要] 背景：异位胃黏膜（HGM）是报道最多的上皮异位，但却极少出现在直肠肛管。

方法：报道了首例内镜黏膜下剥离术（ESD）治疗低位直肠非息肉型 HGM。在 MEDLINE、EMBASE 和 Google Scholar 上，以”直肠和肛管异位胃黏膜”为关键词进行全面检索。

结果：检索到 79 篇文章，经过筛选最终 72 例（包括本例）病例纳入分析。17例（24%）患者具有先天畸形，其中直肠重复畸形占大多数。 HGM 位于肛管和会阴部直肠25例（41%），位于低位、中位和高位盆腔直肠分别为 20 例（33%）、5例（8%）和 11 例（18%）。形态学上非息肉型 37 例（51%），息肉型26例（36%），溃疡型9例（13%）。50例（69%）患者具有特异性肛管直肠症状，其中 34 例≤18岁的患者中有33例（97%）出现特异性症状。23例（32%）患者出现并发症。50 例（83%）患者进行了治疗，其中内镜下切除17例（50%）；在12例≥ 15 mm的病灶中5例（42%）成功进行了分块切除，2例（17%）需行氩离子凝固治疗，2例（17%）术后发现HGM残留。

结论：本系统综述显示直肠肛管 HGM 具有特异性直肠症状，而且会出现一些严重并发症，主要是对于小儿患者，成年患者则有一定的恶变风险。推荐进行完整切除，ESD 可以克服传统套圈进行完整切除切除的技术局限，从而避免外科手术。

关键词：异位胃黏膜；内镜黏膜下剥离术；综述；直肠

*This article abstract was originally published in English. This translation has not been verified and should not be relied upon—it is provided for reference purposes only. The Publishers have not checked this translation and accept no liability for completeness or accuracy of this translation or the use of this translation for whatever purpose. This translation may be incomplete and inaccurate in whole or in part. If you need to rely upon a translation of this abstract, a professional human translator should be engaged to supply an accurate translation of the original English. When referencing articles from this journal, please always refer to the original English version, rather than a translated equivalent.
本文摘要最初是以英文发表。中文摘译未获认证，谨供参考。出版社没有对中文摘译进行核验，不负责译文的完整性和准确性，也不会将其用以其他目的。中文摘译可能会出现部分不完整或不准确的情况。如果你需要准确获悉摘要，最好能请一位专业译者针对英文原文进行翻译。阅读杂志文章，请参考英文原文，不推荐中文翻译。

### Commentary （评述）

### Neoadjuvant treatment of rectal cancer: where are we now?
### 直肠癌的新辅助治疗：我们目前身处何处？

### Original article （论著）

### Non-surgical porto-mesenteric vein thrombosis is associated with worse long-term outcomes in inflammatory bowel diseases
### 非手术相关的门静脉/肠系膜静脉血栓形成可致炎性肠病患者长期结局不良

[摘要] 目的：本研究旨在评估炎性肠病（IBD）患者出现非手术相关的门静脉/肠系膜静脉血栓形成（PMVT）的危险因素及其对患者长期结局的影响。

方法：通过电子医疗记录纳入 2004﹣2013 年间 20 例 IBD 合并 PMVT 患者（研究组）。根据年龄、性别及 IBD 分型进行 1:3 匹配，筛选出 60 例未合并 PMVT 的 IBD 患者（对照组）。分析 PMVT 的危险因素，并比较 PMVT 诊断后 1 年两组患者 IBD 相关结局。

结果：研究组 20 例患者中溃疡性结肠炎 6 例（30%），克罗恩病 14 例（70%），其中11例（55%）为男性。多因素分析显示，住院治疗（OR=6.88; 95%CI: 1.88﹣25.12）和甾体类药物使用（OR=4.39; 95% CI: 1.27﹣15.19）是PMVT形成的独立危险因素。随访 1 年，PMVT患者 IBD 相关结局较非 PMVT 患者更差，包括后续的急诊看病率（26.3% vs. 1.7%; P=0.003），入院治疗率（60.0% vs. 20.0%; P=0.001），IBD 相关手术率（65.0% vs. 26.7%; P=0.003）。多因素分析显示，PMVT（OR=5.19; 95%CI: 1.078﹣25.28）和住院治疗（OR=8.92; 95%CI: 1.33﹣59.84）是预后不良的独立危险因素，而免疫抑制剂的使用则是一项保护因素（OR=0.07; 95%CI: 0.01﹣0.51）。

结论：住院治疗或接受甾体类药物治疗的 IBD 患者出现 PMVT 的风险增高。PMVT 的发生可导 IBD 患者临床结局不良。

关键词：炎性肠病；门静脉血栓形成；临床结局；危险因素

### Predicting morbidity and mortality in acute pancreatitis in an Indian population: a comparative study of the BISAP score, Ranson's score and CT severity index
### BISAP评分与Ranson评分和CT严重指数预测印度急性胰腺炎患者并发症发生率和病死率的比较研究

[摘要] 目的：本研究通过印度中东部地区一家三甲医院，前瞻性评估急性胰腺炎严重度床边指数（BISAP）评分预测急性胰腺炎患者病死率及疾病进展中间指标的准确性，从而为贫困人群提供一个经济、有效的预后预测方法。

方法：2012﹣2014年间我们中心连续收治的 119 例急性胰腺炎患者纳入研究。起病 24 h内对所有病例进行BISAP评分，同时也进行Ranson评分和 CT严重指数(CTSI) 评估。采用趋势和判别分析来对这三种评分系统对病死率的预测价值进行评价。通过受试者操作特征（ROC）曲线评估这三种评分系统对急性胰腺炎患者病死率及疾病进展中间指标（器官衰竭和胰腺坏死）的预测情况。

结果：119例入组病例中 42 例（35.2%）出现器官衰竭，定义为重症急性胰腺炎，47例（39.5%）发生胰腺坏死，12例（10.1%）死亡。BISAP评分预测重症急性胰腺炎、胰腺坏死和死亡的曲线下面积（AUC）分别为 0.962、0.934 和 0.846。Ranson 评分预测重症急性胰腺炎（AUC 0.956）和死亡（AUC 0.841）的准确性稍低于 BISAP 评分。CTSI在预测胰腺坏死方面的准确性最高，AUC为0.958。以≥ 3 分作为截点，BISAP评分预测病死率的敏感性和特异性分别为100%和69.2%。

结论：BISAP 评分简单实用，在急性胰腺炎起病24 h内即可鉴别出高危死亡风险及高危疾病进展风险的患者。这种风险分层方法可用以提高临床诊治，并有助于临床研究中的病例入组。

关键词：急性胰腺炎；病死率；BISAP评分；Ranson评分；CT严重指数（CTSI）

### Radiographical findings in patients with liver cirrhosis and hepatic encephalopathy
### 肝硬化合并肝性脑病患者影像学表现

[摘要] 背景与目的：肝性脑病是肝硬化患者的常见并发症，而肝性脑病患者目前并不适宜进行肝移植。本研究旨在明确影像学提示的肝性脑病与患者临床特征的关系。

方法：回顾性分析 2009﹣2010 年间进行肝移植评估的肝硬化患者。排除肝癌、射血分数不足60%、曾行经颈静脉肝内门体分流术以及未完成评估的患者。单因素分析有统计学意义（P<0.05）的变量纳入多因素逻辑回归模型。

结果：共计117例肝硬化患者满足纳入标准入组研究，其中肝性脑病组58例，对照组 59 例。单因素分析显示，门静脉直径较小、肝前后径较短、肝结节、应用利尿剂/中枢作用药物与肝性脑病的发生密切相关。多因素分析证实了门静脉直径和应用利尿剂/中枢作用药物与肝性脑病的关系。

结论：门静脉直径缩小提示肝性脑病的风险增加。找出门静脉直径较小的肝硬化患者，通过进一步的心理学测试来对肝性脑病进行筛查，并采取更积极的措施来处理便秘及其他易导致肝性脑病的因素。

关键词：肝性脑病；肝硬化；门静脉直径；CT

### Primary sclerosing cholangitis and the risk of colon neoplasia in patients with Crohn's colitis
### 原发性硬化性肝管炎与克罗恩病患者结肠瘤变的风险

[摘要] 背景与目的：克罗恩病结肠炎（CC）常伴发原发性硬化性肝管炎（PSC）。然而，在CC/PSC患者中结肠癌或瘤变的风险仍不清楚。本研究目的是研究伴或不伴PSC的CC患者结肠瘤变的风险。

方法：本研究系巢式病例对照研究，研究对象是 1985﹣2012 年间 Cleveland Clinic 收治的 CC 合并 PSC 患者。比较 43 例 CC 合并 PSC 患者（PSC组）与同期 159 例 CC 未合并 PSC 患者（对照组）的结肠瘤变率。

结果：43例PCS组患者中有7例（16.3%）出现结肠癌或瘤变，而159例对照组中有 22 例（13.8%）出现结肠癌或瘤变（P=0.98）。PSC组出现结肠瘤变的7例患者瘤变均发生在近端结肠（脾曲近端），而对照组仅50%（11/22）的瘤变发生在近端结肠（P=0.001）。Cox回归分析显示，男性患者（HR=2.68; 95%CI: 1.30﹣5.54; P=0.008）和CC诊断年龄（HR=1.29; 95%CI: 1.14﹣1.47; P<0.001）是发生结肠瘤变的独立危险因素，而硫唑嘌呤/6巯基嘌呤的使用则具有保护作用（HR=0.30; 95%CI: 0.13﹣0.70; P=0.005）。合并PSC并不会增加结肠腺瘤的发生风险（HR=0.45; 95%CI: 0.18﹣1.13; P=0.09）。

结论：合并PSC并不会增加CC患者结肠瘤变的风险。在我们这组CC/PSC病例中，结肠瘤变均发生在近端结肠。

关键词：克罗恩病；原发性硬化性胆管炎；结肠瘤变

### Endoscopic management of refractory gastrointestinal non-variceal bleeding using Histoacryl (N-butyl-2-cyanoacrylate) glue
### 组织粘合剂（N-butyl-2-cyanoacrylate）应用于顽固性非静脉曲张性胃肠出血的内镜治疗

[摘要] 目的：组织粘合剂（N-butyl-2-cyanoacrylate）在胃肠静脉曲张出血的应用价值已经得到了良好的体现，但其在非静脉曲张出血中的应用情况却不甚明了，也鲜有报道。

方法：对6例顽固性胃肠非静脉曲张出血患者实施组织粘合剂注射止血。所有 6 例患者经保守止血和/或介入栓塞治疗无效，且由于严重的合并症或麻醉风险不宜行急诊手术。内镜止血采用”碘化油-组织粘合剂-碘化油”三明治注射技术。评估这些病例的临床结局和并发症。

结果：6 例患者中女性4例，男性 2 例，平均年龄 55 岁。出血原因包括胃溃疡2例，十二指肠溃疡 2 例，十二指肠胃肠间质瘤 1 例，直肠溃疡 1 例。所有患者经组织粘合剂注射均成功止血，无需补救性手术，亦无治疗相关的并发症和死亡发生。2例患者在初次组织粘合剂注射后仍有出血，经再次组织粘合剂注射成功止血。

结论：组织粘合剂止血应作为顽固性非静脉曲张性胃肠出血的一种治疗选择。

关键词：组织粘合剂；N-butyl-2-cyanoacrylate；内镜；非静脉曲张性胃肠出血

### Perspectives of physicians regarding screening patients at risk of hepatocellular carcinoma
### 医生对肝癌高危患者筛查的态度

[摘要] 背景与目的：对具有肝癌风险的患者进行筛查有助于肝癌的早期诊断，从而改善预后。肝癌最常见的病因包括慢性病毒性肝炎（如乙肝、丙肝）和肝硬化。本研究目的是评估医生对肝癌高危患者筛查的认识。

方法：来自3个医疗中心的 300 名医生受邀参加了此次邮件问卷调查。主要结局指标是医生对目前的肝癌筛查指南的认识。通过问卷收集人口统计学资料及临床资料。

结果：300名受邀的医生中有177人（59.0%）完成了问卷，其中机构医生129人，住院医生46人，规培医生2人。按照专业，内科医生占62.1%，家庭医生占16.4%，胃肠专科医生占15.3%，肿瘤科医生占3.4%，其他2.8%。分别有163名（92.1%）和167名（94.4%）的医生会对由慢性乙型和丙型肝炎继发的肝硬化患者进行肝癌筛查；其中35%的医生采用每6月一次的甲胎蛋白（AFP）检测进行筛查，22.0%的医生采用每6月一次的影像学筛查。而有22名（12.4%）医生不会进行血清AFP检测，33名（18.6%）医生从未使用过影像学筛查。

结论：大多数参加调查的医生都会对高危人群进行肝癌筛查，但最合理的筛查方法（如影像学检查）并没有为大多数医生采用，他们更多的是依赖 AFP 的检测。

关键词：肝癌；筛查；慢性肝病；肝硬化

### Case Report （病例报告）

### Mediastinal pancreatic pseudocyst with hemorrhage and left gastric artery pseudoaneurysm, managed with left gastric artery embolization and placement of percutaneous trans-hepatic pseudocyst drainage
### 采用胃左动脉栓塞加经皮经肝假性囊肿抽吸治疗纵隔胰腺假性囊肿出血合并胃左动脉假性动脉瘤

### Inguinoscrotal region as an unusual site of extra-pancreatic collections in infected pancreatic necrosis
### 一处罕见的感染性胰腺坏死的肝外液体积聚区：腹股沟阴囊区

### Endoscopic retrieval of gastric trichobezoar after fragmentation with electrocautery using polypectomy snare and argon plasma coagulation in a pediatric patient
### 采用息肉圈套切除和氩离子凝固进行电碎裂后内镜下取出儿童胃内毛石

### An unusual cause of acute anemia in an immunosuppressed patient
### 一例免疫抑制患者出现急性贫血的罕见原因

### Endometriosis mimicking colonic stromal tumor
### 子宫内膜异位症误诊为结肠间质瘤
